# Characteristics of Participants Enrolled in a Brief Motivational Enhancement for Smokers

**DOI:** 10.3389/fpsyt.2016.00077

**Published:** 2016-05-02

**Authors:** Amy L. Copeland

**Affiliations:** ^1^Department of Psychology, Louisiana State University, Baton Rouge, LA, USA

**Keywords:** motivational interviewing, smoking cessation, smoking, nicotine, addiction and addiction behaviors

## Abstract

Daily smoking is associated with elevated blood pressure, carbon monoxide (CO) toxicity, and impaired pulmonary lung functioning. The benefits of successful smoking cessation are readily apparent, given the health improvements associated with cessation, as well as the reduction of secondhand smoke to which non-smoking coworkers and family members are exposed. Previous literature indicates that providing personalized information to smokers (versus general base rates) without engaging in confrontational pressure to quit smoking, leads to increased interest in quitting smoking and willingness to enter smoking cessation programs. The goal of this study was to examine the pretreatment characteristics of the smokers entering a brief motivational enhancement intervention based on personally tailored health feedback. Participants (*N* = 28) were 88.2% Caucasian and 59% males, and they were an average of 23 years of age. On average, they smoked 20.08 cigarettes per day for a mean of 6.6 years, a mean Fagerström Test for Nicotine Dependence score of 4.7, and obtained a mean breath CO reading of 19.1 ppm. Smoking-related adverse health outcomes were predictive of stages of change motivation to quit smoking. Implications for cessation programs are discussed.

## Introduction

Despite the widely publicized negative health consequences of cigarette smoking, a quarter of the United States adult population continues to smoke (1). Cigarette smoking remains the leading preventable cause of death in the United States, accounting for more than 430,000 deaths per year (2). Smoking cessation is associated with decreased mortality and morbidity from smoking-related illnesses, such as cancer. For example, former smokers reduce their excess lung cancer risk by 50–80% within 10 years of quitting (3).

Research indicates that effective interventions exist for cigarette smokers (4) and that as many as 60% of current smokers want to quit smoking (5), yet few enter formal smoking cessation treatment programs [e.g., Ref. (6)]. Recent studies have also indicated that among current smokers, nicotine dependence levels tend to be higher than in past years. In addition, the prevalence of comorbid psychopathology, such as depression and anxiety, in smokers is greater now than in previous years, because those without such difficulties have largely been able to quit smoking [e.g., Ref. (7, 8)]. As a result, prevalence for those with psychiatric disorders has remained disproportionately high (9, 10). These smokers tend to experience more difficulty with smoking cessation and maintaining abstinence, and are therefore likely to require and benefit from professional assistance in quitting smoking. This suggests that cessation efforts need to be available and readily accessible (e.g., workplace; primary care settings) in order for more smokers to take advantage of these resources. Smokers with few resources and of diverse ethnic backgrounds continue to be underrepresented in cessation programs. In a previous study (11), smokers identified transportation and other practical issues among smokers who were socioeconomically disadvantaged. There were also reports of disbelief that smoking was personally harmful to the individual.

Motivational interviewing (MI) (12) is a clinical intervention procedure in which a collaborative, cooperative alliance is formed between the therapist and the individual suffering from an addictive disorder, such as smoking/nicotine dependence. Contrary to the traditional clinical approaches with substance users in which the individual is forced to label him/herself as an addict or be declared in “denial” of his/her addiction, the MI approach elicits motivation from the substance user to change by querying in a non-confrontational manner about adverse consequences the addiction has caused in the individual’s life, assists the substance user in weighing the pros and cons of continued use, and offers a variety of intervention suggestions if solicited from the substance user. This non-judgmental, collaborative approach has been shown to be effective in assisting substance users/smokers to become more “ready” to change their substance use behavior. Prochaska and DiClemente (13) discuss this “readiness for change” in health behaviors as occurring in various stages, ranging from precontemplation, contemplation, preparedness, action, and relapse. During each of these stages, individuals are more open or “ready” to accept particular treatment interventions than others. That is, an individual in precontemplation (defined as not believing that his/her substance use is even problematic) would not be receptive to specific strategies for ceasing to use a substance, whereas, an individual in the preparedness or action stage would be receptive to structured suggestions of this type, having already made the decision to stop using the substance and in need of concrete skills. According to contemporary models of high-risk behavior change, in addition to appreciating that risks associated with the behavior apply to him/herself, an individual must view the barriers to quitting that behavior as surmountable, in order to cease participation in that high-risk behavior [e.g., Ref. (14)]. The MI component of offering a variety of intervention suggestions to the individual, if solicited by him/her, could supply this information to the smoker. This would include the fact that there are many efficacious and effective interventions that presently exist for smokers (4).

In the present study, daily smokers were entering an intervention which utilized the feedback component of MI with smokers who were not yet “ready” to quit smoking (13) by agreeing to participate in a paid study entitled, “*Health Screen for Smokers*.” We were interested in examining the pretreatment characteristics of these smokers; in particular, what characteristics would be most associated with present motivation for smoking cessation.

## Materials and Methods

### Participants

Participants were Louisiana State University campus employees recruited through fliers on campus and advertisements on their paystubs for a paid study entitled, “*Health Screening for Smokers*.” They were randomly assigned to the active treatment condition, in which they obtain personalized information regarding their blood pressure, carbon monoxide (CO) level, and pulmonary lung functioning, or the control group in which they underwent the health screening, but they received no feedback. All participants were offered smoking cessation treatment free of charge at the campus clinic. Outcomes from this motivational intervention are not reported here. Rather, we examined smokers’ pretreatment characteristics.

### Instruments

#### Demographic Questionnaire

This form assessed participant demographics, including age, gender, ethnicity, education level, occupation, and income. We also included questions regarding medical insurance status and smoking-related illness.

#### Smoking Status Questionnaire

This form assessed current and past smoking patterns and included the Fagerström Test for Nicotine Dependence (FTND) (15). The FTND yields scores that range from 0 to 10. This form also included a question regarding previous smoking cessation attempts and the questions, “*Have you experienced any smoking-related health problems*?” and “*If so, please indicate the category that best describes the health problems you’ve experienced*,” with the response options of “respiratory,” “circulatory,” “cardiac,” “cancer,” or “other.”

#### Stages of Change Algorithm

The stages of change (SOC) algorithm (16) was used in order to obtain a categorical measure of participants’ stage of change. This form comprised a series of mutually exclusive questions: (1) “Are you seriously considering quitting smoking in the next 6 months?”; (2) “Are you planning to quit in the next 30 days?”; and (3) “In the last year, how many times have you quit for at least 24 h?” in order to identify participants as being in precontemplation, contemplation, or preparation. This measure may be viewed as an indication of how ready a smoker is to quit smoking.

#### Brief Smoking Consequences Questionnaire-Adult

This questionnaire measures smoking outcome expectancies, anticipated rewarding, and punishing consequences from smoking a cigarette (17). The Brief Smoking Consequences Questionnaire-Adult (BSCQ-A) comprises 10 factors derived from principal components analysis: (1) negative affect reduction; (2) stimulation/state enhancement; (3) health risks; (4) taste/sensorimotor manipulation; (5) social facilitation; (6) weight control; (7) craving reduction/addiction; (8) negative physical feelings; (9) boredom reduction; and (10) negative social impression. Scores on each of the 10 scales are calculated by taking the average of the items. Scale scores range from 0 to 9.

#### BreathCo Carbon Monoxide Monitors (Vitalograph, Inc.)

The BreathCo CO monitors were used to determine expired CO level (ppm). A cutoff of >8 ppm was used to confirm daily smoking status.

#### Lung Age Meter

A key indicator of chronic obstructive pulmonary disorder (COPD) is a reduced FEV1 compared with predicted FEV1 value. Early identification of a reduction in FEV1 can provide early warning of the damage already suffered by the lungs at the pre-symptomatic stage, when smoking cessation is most effective. The Vitalograph lung age compares a subject’s FEV1 with predicted normal values to calculate the subject’s “lung age.” A high lung age in relation to the subject’s chronological age can illustrate the likely negative impact of continued smoking on lung function and encourage smoking cessation.

### Procedure

Participants called in response to an advertisement for paid research for smokers in a study entitled, “*Health Screen for Smokers*.” They completed a brief phone screen with a member of the research team to determine basic eligibility [>18 years of age; self-report of current smoking rate >20 cigarettes per day (CPD) for at least 1 year], listened to a brief description of the study, including the provision of health-related feedback on CO and lung functioning, and were told that they would receive $50.00 for completion of the study. They were scheduled for a session at which they were told their smoking would be biochemically verified. They were greeted by a research assistant when they arrived and were randomly assigned to the active treatment condition, in which they obtain personalized information regarding their CO level and pulmonary lung functioning, or the control group in which they underwent the health screening, but they received no feedback. All participants were offered smoking cessation treatment free of charge at the university campus clinic. Participants completed the questionnaires and the research assistant obtained breath samples for the CO reading and for the lung age meter reading. Participants were paid $50 for their participation. All study procedures, including the ethical treatment of human subject participants, were reviewed and approved of by the Institutional Review Board of Louisiana State University.

## Results

### Participant Characteristics

Participants (*N* = 28) were 88.2% Caucasian and 59% males, and they were an average of 23 years of age. On average, they smoked 20.08 CPD for a mean of 6.6 years, a mean FTND score of 4.7, and obtained a mean breath CO reading of 19.1 ppm. Participants’ responses to the SOC measure indicated that 47% were in pre-contemplation and 35.3% were in the contemplation stage.

Forty-three percent of participants endorsed having a diagnosed smoking-related health problem, of which 75% were identified as respiratory and the rest as other than respiratory, circulatory, cardiac, or cancer. The mean lung age meter reading among participants was 65.9 (SD = 29.6), and the mean scale score on the BSCQ-A for health risks was 8.3 (SD = 5.6).

### Prediction of SOC Readiness to Change

We used linear regression analyses with health indices as predictors and SOC readiness to change as the dependent variable. Currently, having a smoking-related health problem significantly predicted SOC readiness to change, such that smokers with a smoking-related health problem reported themselves as more “ready” for smoking cessation (β = 0.35), *F*(1, 27) = 3.56, *p* = 0.035; *R*-squared 0.125, adjusted *R*-squared 0.09. Regression analyses with BSCQ-A health risk scale scores and lung age were not significant in predicting SOC readiness to change.

## Discussion

Participants in the present study were part of a larger study in which personally tailored health feedback was provided to daily smokers utilizing MI techniques. In the present study, we were interested in examining pretreatment characteristics among daily smokers, including their smoking patterns, smoking outcome expectancies, and smoking-related health information. This included their CO levels and lung age related to risk for COPD.

Smokers in this study were predominantly Caucasian, young adults. On average, they smoked one pack per day of cigarettes for approximately 7 years and were moderately dependent on nicotine, as indicated by their FTND scores. They endorsed strong beliefs regarding the health risks associated with smoking, as reflected on their scores obtained on the health risks scale of the BSCQ-A. In fact, these health risk scores were sufficiently high at pretreatment that a ceiling effect may preclude detection of pre- to posttreatment increase in the tailored feedback condition of the larger intervention study. A similar finding in a smoking expectancy challenge with health risk expectancies suggests that current smokers identify the health risks associated with smoking, but that these beliefs can be increased by providing health information, and that this increase in expectancies may be accompanied by increased motivation to quit smoking (18). In addition, in the development study for the Smoking Consequences Questionnaire-Adult (SCQ-A), health risk expectancy scores significantly distinguished between smokers entering cessation treatment and those not currently interested in cessation (19). These findings within the context of previous literature therefore support the utility of targeting health risk expectancies in attempts to increase motivation for smoking cessation.

The present study suggests that recruitment of precontemplators was successful, in that almost half (47%) of the smokers who agreed to participate were in the precontemplation stage of readiness to quit smoking. This goal was consistent with the larger study’s goal of increasing motivation to quit smoking (e.g., progression from precontemplation to contemplation) by providing tailored, smoking-related health feedback to participants. Participants who were already motivated to quit smoking in the present study were more likely to endorse having a smoking-related health problem. Given this finding, it will be interesting to see whether tailored health feedback to participants regarding their CO level and pulmonary functioning/lung age is associated with increased motivation for cessation. Such results would be consistent with interventions that increase progression in stage of change, according to Prochaska and DiClemente (13). That is, an individual in precontemplation would be receptive to strategies that increase his/her awareness of the adverse effects of smoking. Further, the MI component of providing treatment information should assist the individual in viewing the barriers to quitting smoking as surmountable. According to Reyna and Farley’s model (14) of high-risk behavior change, this is a critical step in addition to the individual appreciating that risks associated with the behavior apply to him/herself.

There are several limitations to the present study that should be noted. First, the study is cross-sectional and therefore all results are correlational in nature. We have explicitly stated that the purpose of this brief study was to examine and describe pretreatment characteristics, and it was anticipated that these characteristics may assist in informing cessation interventions. The present study was conducted as a portion of a larger experimental study, which includes a motivational intervention for daily smokers based on tailored health feedback. Second, the sample size is small, which may limit generalizations that can be made to other populations of daily smokers. Finally, the participants in the present study were young adults with an average age of approximately 20 years, which may further compromise the study’s external validity in that typical smoking cessation treatment participants are older and may differ significantly from the present sample in other important ways as well. Indeed, the study was designed with mature, adult smokers in mind, and the adverse health information provided to participants would likely be more severe if this population had been recruited into the present study.

## Author Contributions

The author confirms being the sole contributor of this work and approved it for publication.

## Conflict of Interest Statement

The author declares that the research was conducted in the absence of any commercial or financial relationships that could be construed as a potential conflict of interest.
